# A simple, dual direct expression plasmid system in prokaryotic and mammalian cells

**DOI:** 10.1093/pnasnexus/pgad139

**Published:** 2023-04-18

**Authors:** Manabu Murakami, Agnieszka M Murakami, Manabu Yonekura, Ichiro Miyoshi, Shirou Itagaki, Yasutaka Niwa

**Affiliations:** Department of Pharmacology, Hirosaki University Graduate School of Medicine, 5 Zaifucho, Hirosaki, 036-8562, Japan; Department of Pharmacology, Hirosaki University Graduate School of Medicine, 5 Zaifucho, Hirosaki, 036-8562, Japan; Department of Pharmacology, Hirosaki University Graduate School of Medicine, 5 Zaifucho, Hirosaki, 036-8562, Japan; Department of Laboratory Animal Medicine, Tohoku University School of Medicine, 2-1 Seiryo-machi, Aoba-ku, Sendai, 980-8575, Japan; Collaboration Center for Community and Industry, Sapporo Medical University, S1 W17, Chuo-ku, Sapporo, 060-8556, Japan; Department of Pharmacology, Hirosaki University Graduate School of Medicine, 5 Zaifucho, Hirosaki, 036-8562, Japan

**Keywords:** DNA recombination, fluorescence, mammalian expression vector, plasmid, protein expression

## Abstract

We introduce a simple, dual direct cloning plasmid system (pgMAX-II) for gene expression analysis in both prokaryotic (*Escherichia coli*) and mammalian cells. This system, which uses a prokaryotic expression unit adapted from the pgMAX system and a mammalian promoter, is effective for subcloning using the DNA topoisomerase II toxin CcdB. Given that molecular biological cloning systems broadly rely on *E. coli* for rapid growth, the proposed concept may have wide applicability beyond mammalian cells.

**Significance statement**

We established a simple, universal direct cloning plasmid system for gene expression in both prokaryotic (*E. coli*) and mammalian cells. This system (pgMAX-II) can be applied directly for expression analysis, for example, transient expression in mammalian cells. Because *E. coli* is used for complementary DNA cloning in various species, our system could have wide applicability for expression analyses.

## Introduction

The standard process of creating mammalian transient expression plasmid constructs typically involves two steps: first, the desired gene is subcloned into a subcloning plasmid, such as pBluescript (Agilent Technologies, Santa Clara, CA, USA); then, the desired gene is converted from the subcloning plasmid to a mammalian expression plasmid, such as pcDNA3 (Thermo Fisher Scientific, Waltham, MA, USA; Conversion method) (1). In 2019, we developed a dual (*Escherichia coli* and mammalian) expression plasmid, pgMAX. This system enables efficient subcloning and expression in *E. coli*; moreover, following a simple deletion step of the prokaryotic promoter sequence with the rare-cutter restriction enzymes *Swa*I and *Pme*I and re-ligation, mammalian expression can be achieved (Deletion method) (1).

In the present study, we established a dual expression plasmid (pgMAX-II) that does not require the deletion step of the original pgMAX system. Instead, pgMAX-II can be used as a prokaryotic expression vector with *E. coli* and can also be used directly as a mammalian expression vector (Direct method).

## Results and discussion

### Plasmid construction

**Fig. 1.**
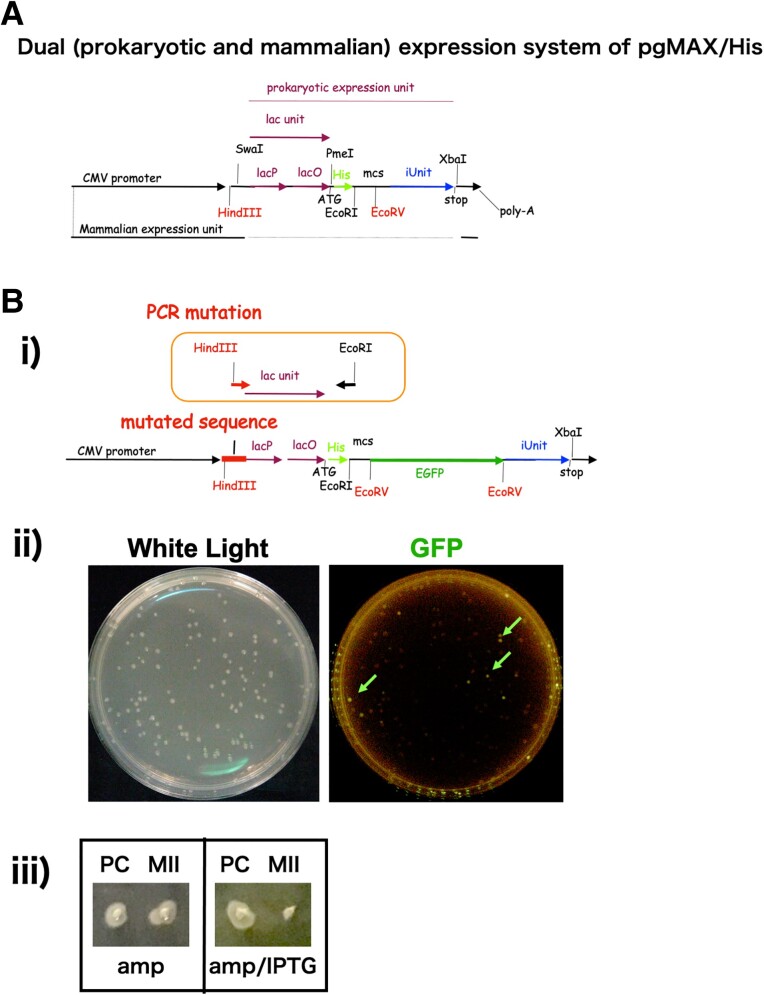
(A) Diagram of the pgMAX/His dual expression system. The *Escherichia coli* expression unit (*Hin*dIII–*Xba*I) consists of a *lac* unit (*lac* operator and *lac* promoter), ATG (start codon), poly-histidine tag sequence, multiple cloning site (mcs, *Eco*RV; GAT′ATC), and inhibitory unit (iUnit; *CcdB*). Insertion of external complementary DNA (cDNA) at the *Eco*RV site leads to the formation of a chimeric gene with the poly-histidine tag, external cDNA, and iUnit. Several restriction sites (*Hin*dIII, *Swa*I, *Pme*I, *Eco*RI, *Eco*RV, and *Xba*I) are indicated. The mammalian expression unit (CMV promoter and poly-A tail) is also indicated. (B) Construction of pgMAX-II. (i) Strategy for *lac* unit mutagenesis. The HindIII-tagged arrow in the inset represents the degenerated oligo DNA (*Hin*dIIInnn-for) used for mutagenesis. The reverse directed arrow represents the specific antisense oligo DNA (*Eco*RI-His-rev). (ii) Detection of *EGFP*-positive clones after mutagenesis. After mutagenesis of the *Hin*dIII–*Eco*RI sequence, fragments were inserted into the *Hin*dIII–*Eco*RI site, transformed, and plated on ampicillin (amp)-containing Luria broth (LB) agar plates with isopropyl-β-d-thiogalactoside (IPTG) induction. The photography light conditions (white light or GFP filter) are indicated. (iii) Confirmation of toxin activity in response to IPTG. On LB agar plates containing amp, both *Escherichia coli* clones with the pgMAX-II plasmid (MII) and *E. coli* clones with pBluescript (positive control; PC) formed colonies (left panel). On LB agar plates containing amp and IPTG, only the PC formed colonies (right panel).

Figure 1A presents an overview of the pgMAX plasmid system (1). The pgMAX plasmid has two functional components: the prokaryotic component for prokaryotic gene expression (*lac* promoter and *lac* operator) with an inhibitory unit (iUnit), containing *CcdB* for efficient subcloning (prokaryotic expression unit, Fig. 1); and the mammalian expression component comprising the cytomegalovirus (CMV) promoter and a poly-A tail (1, 2).

A direct expression plasmid for both prokaryotic (*E. coli*) and mammalian expression requires a sequence that can be used as a prokaryotic promoter in *E. coli* and does not inhibit mammalian gene expression under the CMV promoter.

To develop the pgMAX-II system, the PCR-amplified enhanced green fluorescent protein (*EGFP*) gene was inserted into the *Eco*RV site of pgMAX. Then, the sequence between *Hin*dIII and the *lac* promoter (i.e. the isopropyl-β-d-thiogalactoside [IPTG]-inducible sequence) was mutated by PCR with degenerate oligo DNA (Fig. 1Bi). With this PCR-mutated prokaryotic promoter sequence, GFP fluorescence was observed (excitation wavelength: 470 nm, emission wavelength: 505 nm; Fig. 1Bii). Colonies exhibiting green fluorescence were selected and each plasmid clone was transiently transfected in human embryonic kidney (HEK)293T cells. One clone (out of 16) showed bright GFP fluorescence in both *E. coli* and HEK293T cells, which we named pgMAX-II/EGFP. Then, the *EGFP* gene was eliminated using *Eco*RV. The colonies were inoculated on Luria broth (LB) agar plates containing either ampicillin (amp) alone or amp and IPTG, to confirm the effect of IPTG and toxicity of CcdB protein (Fig. 1Biii). The recombinant group showed no growth on the LB plate containing IPTG, indicative of CcdB expression. This plasmid was named pgMAX-II (DNA alignment of the prokaryotic promoter region of pgMAX and pgMAX-II was shown in the supplementary information).

### Effective subcloning and expression in *E. coli* and HEK cells

**Fig. 2.**
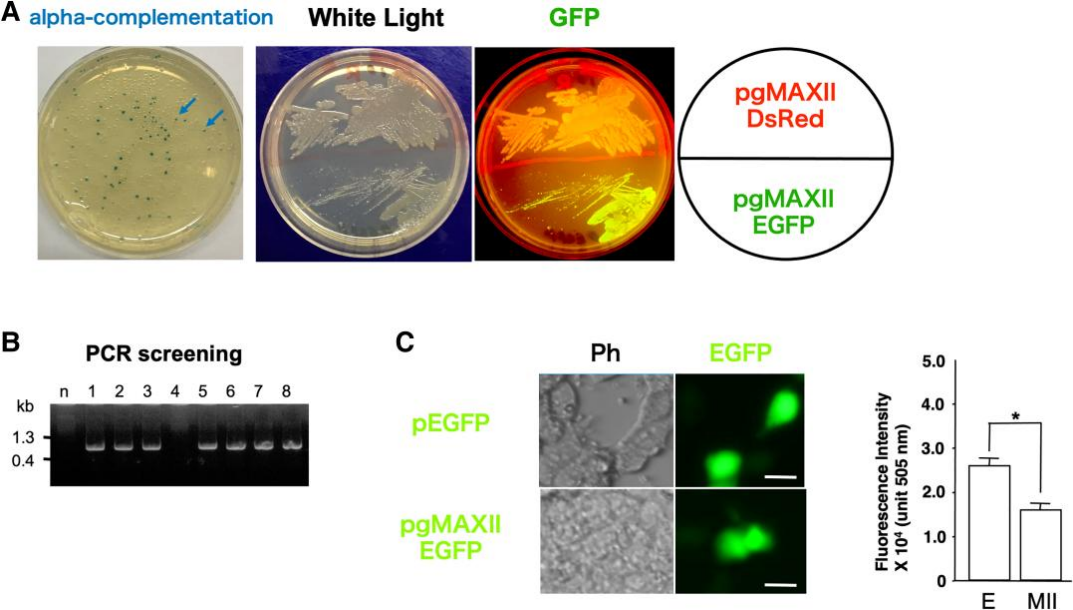
(A) Prokaryotic expression analysis. Blue/white α-complementation selection; arrows indicate examples of blue colonies (left panel). Confirmation of fluorescent protein gene expression (*DsRed2* and *EGFP* in pgMAX-II) using a GFP fluorescence filter (emission wavelength: 505 nm) with blue light (excitation wavelength: 470 nm); the fluorescent protein genes are indicated (right panels). (B) PCR analysis with pgMAX-II/EGFP ligation. Seven of eight (87.5%) clones contained the desired insert; *n* represents the negative control. (C) Direct plasmid expression in human embryonic kidney (HEK) cells. Phase-contrast (Ph) and EGFP fluorescence images of pgMAX-II- and pEGFP-transfected cells (left). Statistical analysis of EGFP fluorescence by pEGFP-transfected cells (E, *n* = 9) and pgMAX-II/EGFP-transfected cells (MII, *n* = 9; *P < 0.05 vs. pEGFP, right panel).

To confirm efficient cloning with pgMAX-II (i.e. successful recombination) and protein expression, the PCR-amplified *α*-peptide sequence of the *lacZ* (β-galactosidase) gene was inserted between the blunt-end sites of *Eco*RV of pgMAX-II (3, 4). Following ligation and transformation, the recombinant clones were plated on LB agar containing amp, X-gal (0.004 mg/mL), and IPTG (for *lac* operon induction). After 16 h, the numbers of blue (*α*-complementation with sense-directed ligation of the DNA fragment) and white (antisense-directed ligation or no insertion of the DNA fragment) colonies were evaluated (Fig. 2A). Nearly one-third (28.4%; 77 of 271 colonies) of colonies were blue, indicative of the high efficacy of the pgMAX-II plasmid for subcloning and protein expression in *E. coli*.

We further examined ligation of the PCR-amplified *DsRed2* fragment (∼700 bp) and *EGFP* gene (∼700 bp). Replated clones containing *DsRed2* or *EGFP* resulted in high expression of the respective fluorescent proteins (Fig. 2A). Figure 2B presents the results of PCR analysis of randomly selected clones after *EGFP* gene ligation in pgMAX-II. PCR analysis revealed successful subcloning (87.5%, 7 of 8 clones contained the desired insert).

### Direct expression in mammalian cells using the pgMAX-II plasmid

To analyze mammalian expression using the pgMAX-II system, we evaluated the pgMAX-II plasmid containing the *EGFP* gene. After 48 h of plasmid DNA transfection in HEK293T cells, EGFP fluorescence was observed under a fluorescence microscope (excitation wavelength: 470 nm, emission wavelength: 505 nm). The pEGFP plasmid (positive control) containing the CMV promoter, *EGFP* gene, and poly-A sequence showed bright fluorescence (Fig. 2C). The pgMAX-II/EGFP plasmid containing the *EGFP* gene in the *Eco*RV site, which showed bright fluorescence in *E. coli*, was used directly (without DNA recombination or deletion of the prokaryotic unit) for transient transfection in HEK293T cells. EGFP fluorescence in HEK293T cells with pgMAX-II/EGFP was comparable with that of pEGFP-transfected cells. Using an ORCA imaging system (Hamamatsu, Shizuoka, Japan) to quantify EGFP fluorescence, the fluorescence intensity associated with transfection with pgMAX-II/EGFP (1.39 ± 0.30 U, *n* = 9) was 52.9% of that with pEGFP (2.63 ± 0.36 U, *n* = 9; *P* < 0.05 vs. pEGFP; Fig. 2C). We also examined pgMAX-II plasmid containing *DsRed2*, and obtained similar results.

In conclusion, we established a simple, universal direct cloning plasmid system for gene expression in both prokaryotic (*E. coli*) and mammalian cells. This system (pgMAX-II) can be applied directly for expression analysis, for example, transient expression in mammalian cells. The highly efficient subcloning of this plasmid system is dependent on the toxicity of CcdB during DNA reproduction in *E. coli* (1, 2, 4). Interestingly, this toxic activity could be easily impacted by externally inserted DNA, resulting in a significant decrease in toxicity and colony formation. Because *E. coli* is used for complementary DNA cloning in various species, our system could have wide applicability for expression analyses.

## Materials and methods

Detailed descriptions are provided in Supplementary Appendix.

### Plasmid construction

The pgMAX-II plasmid containing the *lac* promoter, *lac* operator, and *CcdB* gene was constructed from the previously developed pgMAX plasmid (Fig. 1A) (1). DNA recombination was performed using a standard method. PCR-based mutagenesis was performed to construct the plasmids.

### Cell culture and transfection of HEK293 cells

Cell culture and lipofection were performed with standard method.

## Supplementary Material

pgad139_Supplementary_Data

## Data Availability

All data are contained within the manuscript or Supplementary Material.
