# Supplementary material for: A simple, dual direct expression plasmid system in prokaryotic and mammalian cells
Source: PNAS Nexus. 2023 Apr 18;2(5):pgad139. doi: 10.1093/pnasnexus/pgad139 (PMC10165797; doi:10.1093/pnasnexus/pgad139)
Supplement: pgad139_Supplementary_Data [file pgad139_supplementary_data.docx]

**Supporting Information**

**Strategy of mammalian gene expression**

The traditional method to achieve mammalian transient expression (Conversion method) consists of two steps: subcloning the desired gene into a subcloning plasmid and converting the desired gene from the subcloning plasmid to a mammalian expression plasmid.

It is also possible to construct a mammalian expression vector by inserting a mammalian promoter sequence at the 5′-terminal of the subcloned complementary (cDNA) and poly-A sequence at the 3′-terminal end of the gene (Addition method).

In 2019, we created a dual (*Escherichia coli* and mammalian) expression plasmid, pgMAX, which enables simple and efficient subcloning and expression in *E. coli* (prokaryotic expression mode). In pgMAX, the prokaryotic promoter is deleted using rare-cutter restriction enzymes (*Swa*I and *Pme*I), and re-ligation yields mammalian expression (Deletion method). The pgMAX plasmid enables simple, fast construction of a mammalian expression vector, although steps such as deletion of the prokaryotic expression unit are still required.

Using the novel pgMAX-II, which contains a mutated prokaryotic promoter sequence from pgMAX, the desired gene is subcloned and expressed via isopropyl-β-D-thiogalactoside (IPTG) induction in *E. coli*. With the same DNA construct, the plasmid can also be used as a mammalian expression vector (Direct method).

In the present study, we established pgMAX-II, which is a simple, direct and universal cloning plasmid system, for gene expression in prokaryotic and mammalian cells. pgMAX-II is an improved expression plasmid compared to the original pgMAX system, which has two expression modes: the (subcloning) prokaryotic and mammalian modes. When using pgMAX, the prokaryotic expression unit must be eliminated with the blunt-end-forming rare-cutter restriction enzymes *Swa*I (ATTT′AAAT) and *Pme*I (GTTT′AAAC) to enable mammalian expression [1]. By contrast, pgMAX-II can used directly for both prokaryotic and mammalian expression analyses without elimination of the prokaryotic unit sequence; this offers a major advantage over pgMAX. While pgMAX-II showed decreased expression in HEK293T cells (compared to the fluorescence control groups), it still contains the *Swa*I and *Pme*I recognition sites, which allows for elimination of the prokaryotic expression unit.

It may be possible to apply the novel prokaryotic expression unit of pgMAX-II (*Hin*dIII–*Eco*RI, which contains the *lac* operator and promoter, allowing expression of the cloned gene by the mammalian CMV promoter) to other expression systems. DNA cloning systems typically rely on the rapid bacterial growth of *E. coli*, which has a doubling time of 17 min and can grow 1 × 10^6^ times per 16 h of incubation. Because *E. coli* is used for cDNA cloning in various species, our concept of a dual expression system (*E. coli* and other species) could have wide application beyond mammalian cells. For instance, it may be possible to insert the prokaryotic expression unit into an expression plasmid for fish or other taxa.

Prokaryotic expression systems, such as those using *E. coli*, have been used widely for mass production of recombinant proteins. However, overexpression of proteins in *E. coli* often results in inclusion bodies as a result of protein misfolding and aggregation [2]. To improve the solubility of protein in *E. coli*, several strategies have been used, such as periplasmic secretion, low expression temperature, low IPTG concentrations, and fusion proteins [3, 4, 5]. Thus, it might be possible to overcome this problem by applying the above-described methods.

DNA alignment of E. coli promoter region (pgMAXI and II)

pgMAXI AAGCTTGGTACCGAGCTCGGATCGATAAGTCGACATTTAAATTTTTACGGTTCCTGGGCT 60

pgMAXII AAGCTTGGTACCGAG-----CTCGGATCGATGACATTTAAATTTTTACGGTTCCTGGGCT 55


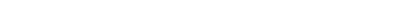
 *************** *** * *****************************

pgMAXI TTTGCTGGCCTTTTGCTCACATGTTCTTTCCTGCGTTATCCCCTGATTCTGTGGATAACC 120

pgMAXII TTTGCTGGCCTTTTGCTCACATGTTCTTTCCTGCGTTATCCCCTGATTCTGTGGATAACC 115


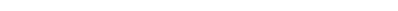


************************************************************


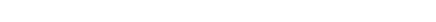


pgMAXI GTATTACCGCCTTTGAGTGAGCTGATACCGCTCGCCGCAGCCGAACGACCGAGCGCAGCG 180

pgMAXII GTATTACCGCCTTTGAGTGAGCTGATACCGCTCGCCGCAGCCGAACGACCGAGCGCAGCG 175


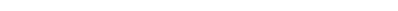


************************************************************


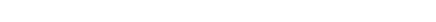


pgMAXI AGTCAGTGAGCGAGGAAGCGGAAGAGCGCCCAATACGCAAACCGCCTCTCCCCGCGCGTT 240

pgMAXII AGTCAGTGAGCGAGGAAGCGGAAGAGCGCCCAATACGCAAACCGCCTCTCCCCGCGCGTT 235


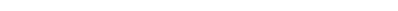


************************************************************


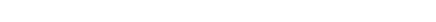


pgMAXI GGCCGATTCATTAATGCAGCTGGCACGACAGGTTTCCCGACTGGAAAGCGGGCAGTGAGC 300

pgMAXII GGCCGATTCATTAATGCAGCTGGCACGACAGGTTTCCCGACTGGAAAGCGGGCAGTGAGC 295


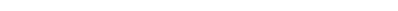


************************************************************


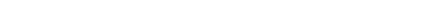


pgMAXI GCAACGCAATTAATGTGAGTTAGCTCACTCATTAGGCACCCCAGGCTTTACACTTTATGC 360

pgMAXII GCAACGCAATTAATGTGAGTTAGCTCACTCATTAGGCACCCCAGGCTTTACACTTTATGC 355


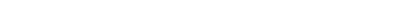


************************************************************


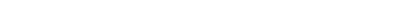


pgMAXI TTCCGGCTCGTATGTTGTGTGGAATTGTGAGCGGATAACAATTTCACAGTTTAAAC 416

pgMAXII TTCCGGCTCGTATGTTGTGTGGAATTGTGAGCGGATAACAATTTCACAGTTTAAAC 411


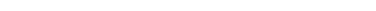


********************************************************

**DNA alignment of the *Escherichia coli* promoter region (pgMAX and pgMAX-II)**

Sequences between *Hin*dIII (A′AGCTT) and *Pme*I (GTTT′AAAC) are shown. Underlining indicates the sequence corresponding to the oligo DNA (*Hin*dIIInnn-for). * symbols indicate identical sequences. The *lac* operator and promotor sequences are indicated by purple and orange text, respectively.

**Materials and Methods**

**Plasmid construction**

The pgMAX-II plasmid containing the *lac* promoter, *lac* operator, and *CcdB* gene was constructed from the previously developed pgMAX plasmid (Fig. 1A) [1]. DNA recombination was performed using a standard method. A blunt-end EGFP DNA fragment was amplified using High-Fidelity Pfu (Agilent Technologies, Santa Clara, CA, USA) with *EGFP*-specific oligo DNA (EcoRVEGFPfor: ccGATATCccggtcgccaccatggtgagcaagggcgaggag; EcoRVEGFPrev: cccGATATCttagggcttgtacagctcgtccat). The PCR-amplified fluorescent genes were inserted into the *Eco*RV site of pgMAX. Following ligation and transformation, the recombinant clones were plated on LB agar containing amp and IPTG (for *lac* operon induction).

PCR-based mutagenesis was performed to construct the plasmids. For mutagenesis at the 5′-terminal sequence within *Hin*dIII–*Eco*RI in pgMAX, *Hin*dIIInnn-for oligo DNA (CCAAGCTTNNNNNNNNNNNNNNNNNNNNNNNATTTAAATTTTTACGGTTCC) and *Eco*RI-His-rev (tcTGAATTCATATGATGATGATGATGATGGGATCCCATGGT) were used.

The conditions for PCR using High-Fidelity Pfu DNA polymerase (Agilent Technologies) were as follows: 25 cycles of denaturation at 98°C for 10 s, annealing at the calculated temperature (~50°C) for 30 s, and extension at 72°C for 30 s. The amplified PCR products were purified using a gel extraction kit (Macherey-Nagel GmbH, Duren, Germany).

**Subcloning and fluorescent protein expression analysis by α-complementation assay**

To confirm DNA recombination and protein expression, a PCR-amplified α-peptide sequence of the *lacZ (*β-galactosidase) gene with EcoRValphaFor (ccGATATCccctatagtgagtcgtatta) and EcoRValphaRev (ccGATATCaggcctccattcgccattcaggctgc) was inserted between the blunt-end sites of *Eco*RV. The host *E*. *coli* strain (K12) harbors the *lacZ* deletion mutation (lacZΔM15), which produces the ω-peptide [3]. Following ligation and transformation, the recombinant clones were incubated on LB agar supplemented with amp, X-gal, and IPTG (for *lac* operon induction). After 16 h, the blue (α-complementation) and white (antisense-directed ligation or no DNA fragment insertion) colonies were counted.

A blunt-end *DsRed2* DNA fragment was amplified using High-Fidelity Pfu DNA polymerase with *DsRed2*-specific oligo DNA (DsRed2for: AaaGCTAGCatgGCCTCCTCCGAGAACGTCATCA; DsRe-d2rev: aaaGAATTCagatctcaggaacaggtggtg).

**Cell culture and transfection of HEK293 cells**

Cell culture and lipofection were performed with standard method. HEK293 cells (ATCC CRL 1573; ATCC, Manassas, VA, USA) were cultured in Dulbecco’s modified Eagle’s medium supplemented with 10% fetal bovine serum. Exponentially growing cells were plated onto 35-mm dishes, and lipofection was performed using commercially prepared lipofectamine (Invitrogen, Carlsbad, CA, USA).

**Statistical analysis**

Data are expressed as mean ± standard error of the mean. After confirmation of a normal distribution with the Shapiro–Wilk test., statistical differences were assessed using Student’s *t*-test, where *P* < 0.05 was considered to indicate statistical significance.

**Supporting Information References**

1. Murakami M, et al. A simple and dual expression plasmid system in prokaryotic (*E*. *coli*) and mammalian cells. PLoS One 2019; 14(5): e0216169. https://doi.org/10.1371/journal.pone.0216169 eCollection 2019. PMID: 31048860
2. Sonoda H, Sugimura A. Improved solubilization of recombinant human growth hormone inclusion body produced in *Escherichia coli*. Biosci. Biotechnol. Biochem. 2008; 72: 2675–2680.
3. Murakami M, et al. A dual prokaryotic (*E. coli*) expression system (pdMAX). PLoS One 2021; 16(10): e0258553. doi: 10.1371/journal.pone.0258553. eCollection 2021.PMID: 34673793
4. Itakura K, et al. Expression in *Escherichia coli* of a chemically synthesized gene for the hormone somatostatin. Science 1977; 198: 1056–1063.
5. Nguyen MT, et al. Prokaryotic soluble overexpression and purification of bioactive human growth hormone by fusion to thioredoxin, maltose binding protein, and protein disulfide isomerase. PLoS One 2014; 9(3): e89038. doi: 10.1371/journal.pone.0089038.eCollection 2014.PMID:24614134
